# Prevention, control, and elimination of neglected diseases in the Americas: Pathways to integrated, inter-programmatic, inter-sectoral action for health and development

**DOI:** 10.1186/1471-2458-7-6

**Published:** 2007-01-17

**Authors:** John C Holveck, John P Ehrenberg, Steven K Ault, Rocio Rojas, Javier Vasquez, Maria Teresa Cerqueira, Josefa Ippolito-Shepherd, Miguel A Genovese, Mirta Roses Periago

**Affiliations:** 1 Area of Health Surveillance and Disease Management, Pan American Health Organization/World Health Organization (PAHO/WHO), 525 23^rd ^Street NW, Washington D.C. 20037, USA; 2 Area of Technology and Health Services Delivery, Pan American Health Organization/World Health Organization (PAHO/WHO), Av. Amazonas 2889 y Mariana de Jesús, Quito, Ecuador; 3 Area of Technology and Health Services Delivery/Area of Legal Affairs, Pan American Health Organization/World Health Organization (PAHO/WHO), 525 23^rd ^Street NW, Washington D.C. 20037, USA; 4 Field Office, US-Mexico Border, Pan American Health Organization/World Health Organization (PAHO/WHO), El Paso, Texas 79912, USA; 5 Area of Sustainable Development and Environmental Health, Pan American Health Organization/World Health Organization (PAHO/WHO), 525 23^rd ^Street NW, Washington D.C. 20037, USA; 6 Veterinary Public Health, Pan American Foot and Mouth Disease Center (PANAFTOSA), Avenida Presidente Kennedy 7778, Sao Bento, Duque de Caxias, 25040-004, Rio de Janeiro, Brasil; 7 Pan American Health Organization/World Health Organization (PAHO/WHO), 525 23^rd ^Street NW, Washington D.C. 20037, USA

## Abstract

**Background:**

In the Latin America and Caribbean region over 210 million people live below the poverty line. These impoverished and marginalized populations are heavily burdened with neglected communicable diseases. These diseases continue to enact a toll, not only on families and communities, but on the economically constrained countries themselves.

**Discussion:**

As national public health priorities, neglected communicable diseases typically maintain a low profile and are often left out when public health agendas are formulated. While many of the neglected diseases do not directly cause high rates of mortality, they contribute to an enormous rate of morbidity and a drastic reduction in income for the most poverty-stricken families and communities. The persistence of this "vicious cycle" between poverty and poor health demonstrates the importance of linking the activities of the health sector with those of other sectors such as education, housing, water and sanitation, labor, public works, transportation, agriculture, industry, and economic development.

**Summary:**

The purpose of this paper is three fold. First, it focuses on a need for integrated "pro-poor" approaches and policies to be developed in order to more adequately address the multi-faceted nature of neglected diseases. This represents a move away from traditional disease-centered approaches to a holistic approach that looks at the overarching causes and mechanisms that influence the health and well being of communities. The second objective of the paper outlines the need for a specific strategy for addressing these diseases and offers several programmatic entry points in the context of broad public health measures involving multiple sectors. Finally, the paper presents several current Pan American Health Organization and other institutional initiatives that already document the importance of integrated, inter-programmatic, and inter-sectoral approaches. They provide the framework for a renewed effort toward the efficient use of resources and the development of a comprehensive integrated solution to neglected communicable diseases found in the context of poverty, and tailored to the needs of local communities.

## Background

### Broader understanding of poverty

Extreme poverty coupled with environmental degradation continues to undermine and circumvent progress toward poverty reduction. The statistics have provided a sobering reality in regards to the current state of affairs in many parts of the world (see Table 1).

**Table 1:** Poverty statistics among the world's population of 6 billion

• Almost 3 billion on less than $2 per day, and 1.2 billion people are estimated to still live on less than $1 per day [1]. In Latin America and the Caribbean (LAC), which has a total population of 561 million [2], 132 million live on less than $2 a day, and 57 million live on less than $1 per day [3]
• 2.4 billion people lack basic sanitation [1]
• 2 billion people are without electricity [4]
• 1 billion adults are illiterate [1]
• 1 billion people are without adequate shelter [4]
• 110 million school-age children are out of school, 60% of them girls [1]
• 1 billion people lack access to safe water [1]
• 880 million people lack access to basic health services [5]
• 790 million people lack adequate nutrition [1]
• 250 million children between the ages of 5 and 14 do wage work outside their household – often under harsh conditions [6]
• One third of human deaths, some 50,000 daily, are due to poverty-related causes and thus avoidable, insofar as poverty is avoidable [7, 8]

Development thinking and practice have evolved in ways that should prove more conducive to tackling the multi-dimensional nature of poverty. Even the understanding of poverty as a concept has broadened. As the Global Poverty Report 2000 makes clear,

"Poverty goes beyond a lack of income. It is multi-dimensional, encompassing economic, social, and governance perspectives. Economically, the poor are not only deprived of income and resources, but of opportunities. Markets and jobs are often difficult to access, because of low capabilities and geographical and social exclusion. Limited education affects their ability to get jobs and to access information that could improve the quality of their lives. Poor health, due to inadequate nutrition and health services, further limits their prospects for work and from realizing their mental and physical potential [9].

### Poverty and conflict

Today, conflict affects some 35 of the world's poorest countries. The uncertainty that comes as a result of living amidst violence erodes social capital and destroys families, creating an environment that makes reintegrating combatants and rebuilding cohesive political systems very difficult [10]. Evidence from case studies and statistical analyses suggests that rising levels of poverty, a decline in state services, and sharp political, social and economic horizontal inequalities between groups are major causes of conflict [11,12]. Environmental degradation, population pressure, falling agricultural productivity and scarcity of water, have also been linked to conflict, as they are sources of poverty [13-15]. These unstable environments are national security threats for both wealthy and poor countries [16].

These threats will continue to rise as poverty is expected to increasingly move from rural to urban areas [17]. The worldwide urbanization of poverty accelerates the risk of instability [18]. This fact becomes particularly noteworthy as the rate of the urban population in developing countries is expected to double over the next 20 years.

### The final straw: disease

The World Health Organization (WHO) estimates that diseases associated with poverty are responsible for 45% of the total disease burden in developing countries [19]. The Millennium Development Goals (MDGs), as well as several other global initiatives have focused exclusively on the control of major communicable diseases with high mortality rates, such as HIV/AIDS, tuberculosis, and malaria. However, this focus has left out a considerable list of "other diseases" that have been aptly coined the "neglected diseases" (NDs). These diseases have been given relatively little attention by national governments and are considered to be low priority international public health issues.

### Neglected diseases

The NDs are largely comprised of infectious tropical diseases. Today, NDs can be usefully considered as a group because they are concentrated almost exclusively among impoverished populations living in marginalized areas. These incapacitating diseases, such as lymphatic filariasis, onchocerciasis, schistosomiasis, soil-transmitted helminthiasis (ascariasis, trichuriasis and hookworm infection), Chagas disease, Buruli ulcer, leishmaniasis, leprosy, and trachoma continue to inflict severe disability and sometimes death. Though the phrase "neglected tropical diseases" is commonly used in the literature today, we have instead chosen the broader phrase "neglected diseases" because some of the infectious diseases of concern in the Americas, such as plague and leptospirosis, are not geographically limited to the tropics and sub-tropics. The NDs also contribute to the overall burden of communicable diseases in the region

Although medically diverse, NDs share features that allow them to persist in conditions of poverty where they frequently overlap [20]. These conditions of poverty include unsafe water, poor sanitation and refuse disposal, which sustain transmission cycles and favor the proliferation of vectors that transmit disease. Other conditions, such as a lack of access to health services, low levels of literacy, inadequate nutrition and poor personal hygiene all help to increase vulnerability to infection and work against prevention efforts. Specific technical opportunities to control NDs in LAC through inter-sectoral and multi-disease approaches were recently reviewed [21], and complement this paper which focuses principally on inter-programmatic opportunities for synergy within health agencies with a focus on PAHO.

### Neglected populations

Neglected populations living in poverty throughout the developing world (e.g., slum and shanty-town dwellers, numerous indigenous groups and small ethnic groups, and the rural poor including migratory workers in agriculture, miners, and fishers) are often heavily burdened by communicable and non-communicable diseases, and highly marginalized by the health sector. In some areas women and children may be considered neglected populations due to their limited access to health and social support services. This hinders their ability to exercise their basic human rights and fundamental freedoms, especially the right to physical and mental health

A reduction in the communicable disease burden would enable these communities and groups to become more economically active and therefore further reduce the socioeconomic factors contributing to disease. A reduction in the total ND burden may not only improve the overall economic performance of families and communities, but of entire nations.

### Millennium Development Goals

In 2000, all 191 United Nations member states unanimously pledged to meet eight MDGs by the year 2015. Among this agenda are the explicit goals of eradicating extreme poverty and hunger (MDG-1), and ensuring environmental sustainability (MDG-7). Communicable diseases are overarching issues of sustainable development rather than exclusively health matters, as evidenced by the high long-term costs, loss of productivity and social burdens associated with illness and disability from NDs, which go beyond the usual economic analysis of ill health.

Although NDs are not explicitly mentioned among the MDGs, the goals cannot be fully achieved without an integrated strategy which includes their prevention, control or elimination. An integrated strategy which includes the NDs supports all eight of the MDGs including ten out of the eighteen Millennium Declaration targets. Several examples of how integrated ND control supports the MDGs are listed below:

• De-worming cost-effectively improves the nutritional status of poor children, contributing to the goal of *Eradication of Hunger (MDG-1) *[22-25], and combines well with vitamin A supplementation.

• De-worming improves school attendance and thus increases the chances of completing primary education, contributing to the goal of *Primary Education (MDG-2) *[22,23,26]

• Promoting income-generating activities such as micro-enterprises for poor women to produce insecticide-treated bed nets, combined with educating mothers in child care and health skills contributes to the *Empowerment of Women (MDG-3) *[27].

• Reducing the combined burden of multiple parasitic diseases (poly-parasitism) [28] and micronutrient deficiencies contributes to the *Reduction of Child Mortality (MDG-4)*.

• Controlling iron deficiency and anemia due to hookworm results in the *Improvement of Maternal Health (MDG-5) *[29].

• Combating NDs contributes to the goal of *Combating HIV, Malaria and other Diseases (MDG-6) *[30] especially where co-infection compounds the health problems of AIDS and malaria victims.

• Implementing environmental sanitation (safe excreta and wastewater disposal) reduces fecal contamination of soil, water, and irrigated crops, contributing to *Ensuring Environmental Sustainability (MDG-7)*.

• Inter-sectoral approaches to ND prevention and control involve establishing extended partnerships compatible with the goal of *Global Partnerships for Development (MDG-8) *[31].

A bold ND prevention and control effort coordinated with other sectors and activities, has the ability to drive the agenda for sustainable development and help achieve the targets for the MDGs. Keeping this objective in mind this paper seeks to examine the following questions:

• How can an integrated ND agenda provide entry points to unify and coordinate the various programs and sectoral agencies involved?

• What are the current PAHO and other institutional initiatives that incorporate and highlight entry points for inter-sectoral initiatives and integrative approaches to health? Are there successful country examples of these initiatives in action?

• What are the common elements that provide a framework for an integrated, inter-programmatic, and inter-sectoral strategy for the prevention, control, and elimination of NDs?

## Discussion: the case for a different approach

### Rationale for an integrated approach

NDs and the environment in which they flourish are intimately tied with other issues of sustainable development, therefore allowing the ND agenda to provide an ideal entry point to develop new paradigms of action. The introduction of basic public health measures in communities would significantly reduce the burden of a number of diseases where these elements play an important role. There is also a considerable overlap in the prevention and management of these diseases, permitting useful synergies amongst these efforts. It is now an opportune time to link an integrated ND strategy with the newly emerging global partnerships addressing HIV/AIDS, tuberculosis and malaria, in order to take advantage of the economies of scale that occur during the scaling up process of these global initiatives [32].

There exists an urgent need to develop innovative tools to combat NDs, particularly ones that move away from a vertical, single disease-centered approach to those that focus on a more horizontal population health approach. The population approach encompasses a broader notion of health which recognizes the range of social, economic and physical environmental determinants that contribute to health. By providing opportunities for integrative solutions to health conditions, and by fostering participatory approaches at the local level, these neglected populations will be better equipped to identify determinants that contribute to poor health, thereby allowing them to exercise their basic human rights, and ultimately break out of the cycle of poverty and illness.

### Integrated, inter-programmatic, and inter-sectoral approaches

Integrated, inter-programmatic and inter-sectoral approaches are not new phenomena. They represent a working multi-disciplinary framework or strategy for which countries, technical assistance organizations and governments should work toward.

The word *'integrated' *stresses that the object or system of interest is a complex, multi-dimensional one, and consists of different interacting elements. These interacting elements may be assessed by examining the available resources within an equity framework. *'Inter-programmatic' *approaches represent opportunities to combine two or more disease-specific or health-specific programs, which are often found in the same health agency.*'Inter-sectoral' *partnering is the process of creating joint inter-organizational initiatives across two or more sectors – inter-sectoral partnerships involve collaboration between organizations that may be based in various sectors: the state (government), the market (business), and civil society (non-governmental organizations (NGOs), non-profits, etc.)" [17].

In the specific context of the ND agenda, integrated, inter-programmatic and inter-sectoral approaches to reach marginalized populations or geographic areas, based on stratification of risks, provide added value for several reasons:

• They represent solutions by "piggy-backing" one disease control intervention with another. For example, combined therapies may be used to control soil-transmitted helminths, schistosomiasis, and lymphatic filariasis by jointly administering praziquantel and albendazole in the same interval [33-35].

• They provide added benefits to the community at large by drawing attention to issues that generally fall outside the purview of the health sector and are intrinsically related with States' human rights obligations (e.g., improved housing and education, provision of clean water, safe disposal of excrement).

• They have the potential to greatly increase the standard of living for the local community by recognizing the economic impact that these diseases have as a result of disability and lost productivity.

Many complex issues, such as housing for the urban poor and local economic development require a wide range of resources and abilities that integration, inter-programmatic and inter-sectoral efforts are the only viable approaches to effectively address them over the long-term and help to reduce the duplication of activities. These approaches can also stimulate innovative solutions by addressing the diverse goals of various participants. In effect, they can produce activities in which "the whole is more than the sum of its parts" [36].

The combination of these approaches are emerging as an increasingly important development strategy. Current trends in the LAC region include a decline in international development funding, the slow decentralization or devolution of national government power and function to municipal and local entities, heightened involvement of the private sector in social issues, and an increasing number of civil society actors [37]. This implies the need for more effective coordination among government programs, increased transparency through participatory approaches and increased access to better information for all stakeholders.

### Opportunities among existing initiatives

Technical cooperation of WHO/PAHO has evolved strategically in direct support to mid-level and local governments and agencies within the framework of decentralization and local development. In particular, there are eight initiatives which provide entry points and opportunities for integration and inter-sectoral partnerships at the local level. They are discussed in more detail below.

They include:

I. Health of the Indigenous Peoples of the Americas Program (PAHO/WHO)

II. Productive and Healthy Municipalities (PAHO/WHO)

III. Community Driven Development (World Bank)

IV. Healthy Municipalities and Communities Initiative (PAHO/WHO)

V. Health-Promoting Schools Regional Initiative (PAHO/WHO)

VI. Primary Environmental Care (PAHO/WHO)

VII. Inter-Sectoral Cooperation: Health and Agriculture (PAHO/WHO)

VIII. Initiative on Public Health and Human Rights (PAHO/WHO)

These initiatives provide a strong foundation to build upon in the LAC region. By examining the overlapping and complimentary features it may be possible to bolster and scale up what has been proven to work, as well as foster future collaborative action.

## I. Health of the Indigenous Peoples of the Americas Program

The Health of the Indigenous Peoples of the Americas (HIPA) Program represents a specific population characterized by precarious health and living conditions, due to an elevated exposure to several factors including; the gradual destruction of the ecosystems supporting their communities, overexploitation of natural resources, natural disasters and the local capacity to respond, labor migration patterns, alcohol abuse, and indiscriminate land colonization by other groups. All of these factors play a significant role in the alarmingly high burden of disease that is ever present within this population. Some communicable diseases that occur with great frequency within this population include malaria, onchocerciasis, acute respiratory infections, tuberculosis and helminthiasis. Additionally, these populations are often plagued with malnutrition, skin infections and diarrhea [38].

In light of the marginalized and underrepresented nature of the indigenous peoples, several attempts have been made to identify strategies that could provide formal health care to this population through an integrated approach while taking into consideration their distinct historical and socio-cultural characteristics. The HIPA Program is a primary example of how integration, inter-programmatic and inter-sectoral activities can provide a synergistic benefit to neglected populations.

The HIPA Program highlights the importance of four lines of work; advocacy and the development of technical capacity and coordination; policy development and targeting for the achievement of the MDGs; information and knowledge management; and primary health care with an intercultural approach.

The program articulates the ability and willingness to incorporate inter-programmatic and inter-sectoral approaches tailored to the needs of specific indigenous populations. The HIPA Program notes that "The establishment of intra-institutional, inter-institutional, and inter-sectoral partnerships has facilitated the incorporation of the health of the indigenous peoples of the Americas into the political agendas and of work within the organization and in institutions that have directives regarding the indigenous peoples of the Region"[38]. Furthermore, the initiative highlights several current integrative and inter-programmatic activities in 14 areas: integrated management of childhood illnesses (IMCI), malaria, tuberculosis, reproductive health, water and sanitation, maternal and child health, virtual campus, mental health, human rights, sexually transmitted infections (STI)-HIV/AIDS, social exclusion, health of older adults, oral health, eye health and rehabilitation [38].

By incorporating a ND component several areas may be highlighted that affect the overall health of specific indigenous populations. As most of these diseases are associated with environmental and behavioral factors, the following areas should be addressed: poor living conditions, unsafe drinking water, inadequate sanitation and excreta disposal, poor drainage, inadequate solid waste removal, poor housing, indoor air pollution and occupational exposure to natural and environmental hazards (e.g, risk of lead and mercury intoxication).

### Water and Parasitic Diseases among Indigenous Populations

High prevalence rates of many parasitic diseases among indigenous populations have been documented [39]. In some indigenous communities, researchers have identified prevalence rates of intestinal helminths as high as 90% [40]. The diseases caused by a scarcity of clean water are the principal causes of morbidity and mortality for indigenous populations [38].

An investigation of the Pankararu indigenous tribe in the state of Pernambuco, Brazil, identified multiple intestinal parasites in nearly all members of the community. Researchers tested relations between daily living conditions (housing, sanitation, water supply/treatment, and garbage disposal) and the number of different parasite species found in the same household. The study concluded that these living conditions had a profound impact on the number of intestinal parasites found among the Pankararu community, with particular emphasis on those relating to lack of adequate water sources and water treatment [41].

Though natural resource development projects have sometimes been linked to the spread or intensification of some parasitic diseases, well-planned development projects which involve local communities in planning and implementation can prove beneficial [42]. For example, efforts in the Peten region and Mayan Biosphere Reserve in Guatemala are a working example of an integrative approach to resource management and indigenous population initiatives fostered by local community participation. In 1988, a strategy for sustainable development for the Peten region was developed by the World Conservation Union (IUCN) at the request of the Guatemalan government. In this particular region of Guatemala, recent changes in land use for the implementation of agro-export commodity schemes have resulted in major climatic and environmental disturbances, changing ownership rights and an overall decline in the quality of life of its indigenous inhabitants [38]. In response to the continual deforestation, inadequate sanitation infrastructure, and diminishing water resources, the Secretary General of Development and Planning (SEGEPLAN) with technical support from a bi-lateral agency (USAID), developed a plan to utilize local participation by resurrecting ancient irrigation techniques for water supply and aquaculture. The project gave primary attention to the incorporation of ancestral technologies of Mayan origin through demonstrative activities that ensure local community participation concerning health, agriculture, and safe and adequate water and sanitation provision [38].

### Education and school health among indigenous populations

Several studies have also examined disparities between indigenous schoolchildren and urban schoolchildren. In a study comparing rural Queimadas Indian schoolchildren with urban schoolchildren in southern Brazil, results demonstrated a strong statistical correlation between stunting and the intensity of soil-transmitted helminth infections among the Queimadas schoolchildren [43]. In a related study, these researchers found that housing/hygiene indicators were significantly poorer for the indigenous schoolchildren, and that there existed a statistically significant positive correlation between total prevalence of soil-transmitted infections and prevalence of high-intensity infections with most variables for poor housing and hygiene. On the basis of these results, recommendations were given to administer mass anti-helminthic treatment in conjunction with educational interventions [44].

### Objectives of the HIPA program

The HIPA Program has several reoccurring themes, such as a call for inter-sectoral collaboration and a renewed effort toward health promotion that "brings together efforts and finds the synergy of actions underway in the countries in achieving the MDGs and the renewal of the primary health care strategy" [38].

The HIPA Program notes, "There is an urgent need for identifying innovative, and at the same time respectful and practical forms [of interaction] to work with the indigenous representatives and to show concrete results whose evidence can be reflected in the reduction of the disease and death in the indigenous communities. This implies the promotion of an integrated work plan that takes into account the conceptions and institutional and community frames of reference and integrates the policies, plans, and action programs considering the strengths, wisdoms, authorities, demands, and processes characteristic of the indigenous peoples within national societies" [38].

These specific action programs in indigenous health represent significant opportunities to reduce the burden of ND on specific neglected populations by considering the comprehensive health determinants that affect indigenous people. This initiative also has the potential to be linked with existing networks of NGOs and other organizations focusing on indigenous populations, which would result in improved environmental sanitation, health education, integrated drug administration, and a focus on nutrition in order to reduce the disproportionate burden of NDs facing these populations.

## II. Productive and Healthy Municipalities Initiative

Poverty rates in rural areas are far higher than in the urban areas, with 64% of the rural population living below the poverty line [45]. The poverty line is calculated according to a 'basic basket' of goods and services that take into account prevailing wage and price structures [45]. Rural poverty disproportionately afflicts women, children and the elderly [46]. Social and economic indicators in rural areas are worse when compared with urban areas. The majority of the indigenous peoples (approximately 80% of 34 million people) located in rural Mexico, Peru, Colombia, Bolivia, Ecuador, and Guatemala are poor [47]. These facts exemplify a close relationship between rural poverty and ethnicity.

Rural development has traditionally been exclusively associated with agriculture. Putting too much emphasis on agriculture and ignoring other aspects of rurality is a pitfall that has been repeated for decades. The policy shift towards integrated rural development reflects the recognition of complex interactions within the system of overall rural development. Integrated rural development provides an alternative to agriculture alone as a source of income and livelihood. This shift represents a fundamental change in policy objectives toward a more holistic and sustainable approach to rurality amongst the most marginalized rural populations [48].

Rural populations face a high-level of social exclusion and social inequity. Many livestock and agriculture products are generated by impoverished and disease-prone workers living in unhealthy environments. With these considerations in mind, PAHO's Productive and Healthy Municipalities (PHM) Initiative articulates an approach to combining agriculture and health in the context of local development to be implemented in rural areas of small livestock producers. This allows for a greater potential to reduce poverty through the improvement of livestock production of small producers [49] combined with rural primary health care.

The PHM Initiative has strong implications for NDs amongst agriculture and livestock producers. The NDs such as Chagas disease and neurocysticercosis are one of the principal causes of morbidity and disability in these populations. The PHM Initiative includes a significant component of health promotion to ensure access to health services for producers and agricultural workers.

The PHM Initiative promotes integrative, inter-programmatic, and inter-sectoral collaboration; "The activities of primary health care should wrap, in addition to the health sector, all the sectors related to local development, in particular to the local government, to the agriculture, production of food, industry, education, housing, public activities, communication, and other sectors and it requires the coordinated efforts of all of those sectors" [49]. This model is based on a systematic approach that integrates the agricultural sector with a rural social structure (including health, environmental sustainability and culture) by emphasizing the importance of "productive family units" [49].

### Effects of microdams and irrigation projects on NDs

Similar to the case of large dams, research in several countries examining the impact of microdams and irrigation projects has shown that these projects can contribute to an increase in favorable environmental conditions for the transmission of parasitic diseases during the dry season, specifically schistosomiasis, intestinal helminths and malaria [50,51]. The number of people living in close proximity to small dams and informal irrigation remains elusive, which inevitably results in an underestimate of the total number of people at risk for parasitic diseases due to water resources development [52]. Health safeguards must be incorporated into the planning, construction and operation of microdams and irrigation systems meant to serve agricultural and livestock producers, as well as larger dams used principally for hydropower and flood control, in order to prevent and reduce these diseases.

For example, recent research on food security and disease transmission suggests that over the last three decades the agricultural-irrigation network has extended globally, thus ensuring water security and increasing the area of arable land that could be farmed by intermittent wet/dry irrigation (IWDI) [53]. As a result of this expansion, malaria vector breeding was proven to have been greatly reduced, representing a significant opportunity for synergy by maximizing agricultural productivity while increasing protective factors for those living in rural communities [54].

### Effects of deforestation and rural colonization

Various economic forces in LAC drive the clearing of forests including cattle ranching, soybean farming, gold mining, hydroelectric dams, and expansion of subsistence agriculture and road construction. In the case of the latter, a recent study in the Peruvian Amazon [55] found that roadside settlements in areas deforested by subsistence farmers experienced up to 278 times higher mosquito-biting rates by the local principle malaria vector *Anopheles darlingi *than those settlements without deforestation. The same area has experienced an upsurge in malaria at the same time, very probably associated with the on-going deforestation. As well, several NDs such as leishmaniasis and Chagas disease are associated with deforestation and rural decolonization. When these diseases are coupled with a lack of access to health services for rural populations it results in a deterioration of the populations health status, further hampering rural agricultural worker productivity and rural family health. As these areas are further developed, strategies to control malaria and other diseases require a combination of preventive and curative methods well as close collaboration between the health and agricultural sectors [56].

### High risk rural populations and protective measures

Malnutrition, diarrhea, anemia and other complications of soil-transmitted helminth infections will often lead to stunting and school absenteeism [57], and probably reduce family economic productivity over the life cycle in both rural and urban areas. High risk rural populations affected by soil-transmitted helminths and other NDs include migrant agricultural workers, itinerant gold miners in Brazil [58], and those living in agricultural labor camps and plantations (e.g., Guatemalan and Mexican coffee pickers with onchocerciasis) [59]. These high disease transmission environments may be mitigated by improved health services including necessary drug treatment, better access to food and micronutrients, and micro-enterprise development to increase incomes which in turn can allow increased individual and family access to health care services. A specific strategy will have to be tailored to the local conditions, partners, community needs, and resources available in the community. Examples of activities that could be integrated in high risk populations include:

• The promotion of household level food production for nutrition and food security, with both de-worming and Vitamin A supplementation [60]

• In trachoma-endemic areas one could add the elements of the trachoma SAFE interventions (Surgery, Antibiotic Therapy, Facial Cleanliness, and Environmental Improvement) with care for skin diseases

• Addressing key micronutrient deficits [61] (e.g., zinc deficiency which is casually associated with diarrhea, pneumonia and malaria in children under age 5 [62]) can be accomplished by adding micronutrients to key foods in the local diet or to condiments such as table salt

• In areas endemic for lymphatic filariasis, diethylcarbamazine (DEC) is added to table salt for mass treatment of at-risk populations and has the potential to eliminate transmission within one to two years. DEC-salt can be combined with iodine and fluoride, as is being utilized in Guyana.

The major challenge ahead is to ensure food security while increasing protective factors for the tens of millions of families living in poverty in LAC. This large and complex task involves increasing agricultural output worldwide, reducing poverty, and improving health and nutrition. These activities have the potential to bolster the productive family units by generating more income and protective factors associated with the rural environment. Developing countries need to improve access to food while also increasing the protective factors of the population by providing education and health services and fostering local participation across sectors.

## III. Community Driven Development

Community participation approaches help to build social capital and prove to be an efficient mechanism for delivering micro-projects which become productive investments. Community participation has been propagated through various initiatives and institutions in development. One such approach is the concept of Community Driven Development (CDD) which the World Bank has intricately linked to various issues of rural and urban services. "Poor people are often viewed as the target of poverty reduction efforts, CDD approaches by contrast, treat poor people and their institutions as initiators, as collaborators and as resources on which to build" [63]. CDD is broadly defined as giving control of decisions and resources to community groups. With a view to generate sustainable and wide ranging impacts, CDD operations and regional strategies have increasingly embraced two important pillars of sustainability and scale: linking communities to the private sector and to local governments.

### Interventions should be tailored to local conditions

Identification of country specific issues is crucial in designing appropriate CDD approaches to development projects and health interventions. In most projects the entry point for local development has often been the project implementation stage rather than the project preparation stage. "In order to enhance the application of CDD approaches in the earlier stages of the project life cycle, additional time and financial resources are needed so that communities can be mobilized and involved in the design and decision making of the overall project framework and components" [63]. By making investments responsible to informed demand, communities are better able to weigh tradeoffs and make realistic choices to fit the local conditions.

The nature of the connection between health and socioeconomic development has become much more evident within the CDD approach. Family health, economic security, environmental sanitation and income generation, all have crucial implications at the local level for combating communicable diseases and the NDs. CDD complements integration, inter-programmatic and inter-sectoral collaboration by emphasizing community ownership and engaging relevant stakeholders in order to garner broad-based support and achieve sustainability.

However, the need remains for greater attention towards the formulation of public policies that will effectively contribute to improving the quality of life for the population, while at the same time promoting equity. In addition, the recent trend of decentralization should lend itself to greater accountability for decision-making and vertical collaboration.

There are several good reasons for the ND agenda to be linked with CDD approaches:

• *Efficiency*: A better fit between program design and community needs that span across sectors through the introduction of basic public health preventive measures, such as education, clean water and sanitation

• *Equity*: Greater community contributions allow marginalized portions of the population to receive information and provide input toward public health decision-making

• *Accountability*: Greater accountability of the programs to communities with greater transparency with mechanisms for local participation built in to institutional design

• *Sustainability*: Greater sustainability because of community ownership through the contribution of local resources for maintenance and improvement

### CDD at work: river blindness in West Africa

An illustration of the CDD approach is the conquest of river blindness in West Africa. River blindness, or onchocerciasis, has virtually been eliminated in 11 countries with a population of 34 million people [64]. This monumental achievement was made possible by country specific CDD approaches that focused on an appropriate division of labor between central governments and local communities. In this instance, large-scale vector control activities were carried out by the government, while local communities managed the distribution of the anti-helminthic drug ivermectin to fight the disease [64]. Community-based ivermectin treatment programs in West Africa supported by vector control have saved the sight of 600,000 people, spared 15 million children from living in at-risk environments, and opened up 25 million hectares of arable land for agriculture [64].

To further ensure institutional sustainability of community based programs, there is a definitive need to link these projects to local governments. PAHO has provided a framework for linking the CDD approach to local governments through the encouragement of health promotion and the Healthy Municipalities and Communities Initiative.

## IV. Healthy Municipalities and Communities Initiative

The Healthy Municipalities and Communities (HMC) Initiative is part of PAHO's Healthy Settings approach and essentially consists of two components; the commitment towards health promotion by local authorities, and the active participation by the community. The strategy encourages health interventions that are highly cost-effective, not only in the case of infectious diseases, but chronic diseases as well. In the HMC Initiative, local development is designed with a focus on building partnerships between local authorities, community leaders and organizations, and private and public sector institutions. Social participation is critical throughout all phases of the process, including the needs assessment, planning, implementation, monitoring and evaluation phases. It helps to create synergy among programs, horizontally and internally within local government structures, and vertically with national and regional priorities. The "Healthy Cities" initiative, which the WHO promoted in Europe and Quebec, Canada, has prompted the countries of LAC to adapt the idea to the local level, municipalities and communities [65].

### Community-based, integrated health promotion

The most successful integrated, inter-sectoral efforts to date have proven to be those that incorporate concrete, community-based initiatives. To be effective, any integration mechanism must place broad-based emphasis on health protection and health promotion [66]. In these environments health promotion serves as the mechanism to build multi-sectoral partnerships and strengthen social participation to upgrade the living and working conditions of the population. This is accomplished with a sustainable process of local planning with health and development targets which are agreed upon among all stakeholders [67]. Thus local development plans respond to the needs and aspirations of local residents, leaders and other stakeholders. The synergy this process creates allows for several targets to be addressed together, rather than developing a plan for each identified issue.

The idea of health promotion in conjunction with community mobilization has particular significance for the ND agenda. By utilizing social participation and community organization, the multi-faceted determinants of disease can be addressed locally through multi-sectoral cooperation. In the case of lymphatic filariasis and soil-transmitted helminthiasis for example, a community will be better equipped to identify and address problems related to unclean water and harmful sanitation practices that are propelling transmission of these diseases. In addition, a more comprehensive base of support initiated through community awareness of the problem, helps to ensure that any health promotion efforts are sustainable over time and across various sectors. In this context, small-scale administrative and political units may provide a more flexible environment for the implementation of inter-sectoral actions, as recommended by HMC.

The main objective of health promotion is to give people greater control over their own health. To achieve this goal, health promotion must transcend the boundaries of the health sector. The health promotion strategy contributes to an improvement in the health status of the population, while simultaneously bolstering activities that mobilize other sectors, such as education. In further recognition of health promotion's critical role in responsive governance, all countries of the Americas signed the Mexico Declaration (Fifth Global Conference on Health Promotion 2000) which embodies a commitment to implement national health promotion plans of action at local and national levels.

### Principal areas of health promotion

The Ottawa Charter for Health Promotion and the Declaration of the International Conference on Health Promotion (the latter held in Santa fe de Bogota, Columbia) identify the following as the principal areas of action for health promotion [68]:

1) The formation of a public health policy that goes beyond the curative dimension, which implies an inter-sectoral view that allows for action on the part of the population, health services, health authorities, and the productive social sectors

2) Creation of environments that will foster good health – in its physical, environmental, and social aspects – through the promotion of healthy communities

3) Strengthening (empowering) of community action in health, since organized community participation facilitates the identification of needs and priorities in order to modify the situation and raise the level of well-being

4) The development of personal skills that give individuals control over their health and environment in order to reduce risk factors for morbidity

5) Reorganization of health services to give priority to health promotion and disease prevention (and tailoring them to specific sociocultural contexts when appropriate)

6) Identification and reduction of the factors that lead to inequity

### Linking local government to health promotion

The HMC Initiative encourages the participation of government authorities and the community through promoting dialogue and fostering collaboration among municipalities and communities [69] and influencing policy development.

HMC strategies encourage the creation of Inter-Sectoral Committees for health promotion in municipalities, with the leadership of the Local Inter-sectoral Committees and the Mayors. These strategies outline provisions for the mobilization of resources, securing adequate support and technical cooperation, and creating healthy and supportive environments in schools, workplaces and public spaces.

### Examples of HMC at work

Current examples of the HMC Initiative at work include efforts in the rural Municipality of Chopinzino, Brazil where inter-sectoral action, combined with strong community participation, helped to broaden the scope of the local council beyond agricultural activities in the rural sector. Under the HMC project the level of education was increased by bringing schools together to improve school transportation, adopting alternative teaching techniques, and a pledge to guarantee education opportunities for all children living in rural areas. In collaboration with the increased dedication to rural education, the project also offered various programs to promote health including: efforts to combat infant mortality, family planning, diabetes prevention and blood pressure monitoring [69].

In the small Canton of San Carlos, Costa Rica the local government initiated the Ecological and Healthy Canton project to bring together various sectors (economic, social, health, education, social welfare, transportation, communications and media) to design a strategy to make the canton a model for health promotion. The project encouraged the active participation of community members in the promotion of health through environmental projects and resulted in greater coverage of environmental education through the media and an "Inter-sectoral Health Fair" held under the slogan of "Protect our Environment" [65]. In such projects, entry points can be created to promote the prevention and control of the NDs of local importance.

It is critical that projects utilizing the HMC Initiative contain a specific ND component. For example, it is estimated that 20 to 30% of the population of the Americas is infected with the intestinal worms *Ascaris lumbricoides, Trichuris trichiura*, and/or human hookworm and *Schistosoma mansoni *[70]. Parasitic worms disproportionately affect children and compete with the child for nutrients, causing anemia and impairing the growth and development of the child, which contributes to a poor quality of life [57]; they also lower the work capacity of adults. Through the involvement of schools and workplaces the HMC strategy can reduce the intensity and prevalence of these parasitic diseases in the community. Evidence demonstrates that the morbidity caused by intestinal parasites can be greatly reduced by comprehensive community-based programs. Management of these programs can be established with control activities being undertaken through existing health facilities and the education sector. The strategy is based upon the integration and inter-sectoral efforts to deliver periodic chemotherapy (once or twice a year depending upon the prevalence and worm burden in the area) to schoolchildren in high-risk areas, intense health and hygiene education, and improvement of sanitation and a safe water supply. In one particular study, the results of this method demonstrated an overall reduction in prevalence of parasitic infections of 44%, illustrating the HMC strategies potential to drastically reduce the burden of parasitic diseases among specific populations. By incorporating a ND component in health promotional activities the community will be better sensitized to the problem and its determinants which can begin to break the cycle of ill-health and poverty.

The HMC Initiative represents an excellent opportunity to coordinate the needs identified by the local community within the broader framework of health promotion. Under this strategy, health promotion acts as the vehicle for linking the various sectors (i.e., environment, agriculture, health, education) for the common goal of promoting health and addressing the underlying determinants that govern health.

## V. Health-Promoting Schools Regional Initiative

The Health-Promoting Schools (HPS) Regional Initiative is also part of PAHO's Healthy Settings approach. As such, it advocates for Health Promotion strategies in the school setting to improve the health and well-being of students and the school community, including teachers, families and the surrounding school population.

Today children, adolescents, and young people require an education for life aimed at the development of their innate capacity to learn to be, learn to learn, learn to do, learn to live with others, as well as to learn to undertake actions. For this, there is the need for the implementation of participatory education to develop students' analytical and inquiring capacity, and to strengthen their principles of respect for human rights, equity, and collective values.

Schools have the responsibility for the implementation of health related activities. Traditionally, these activities have been characterized by *ad hoc *efforts, mostly directed to improving conditions of hygiene and environmental sanitation, preventing communicable diseases, treating specific diseases, and performing sporadic medical examinations or screening tests. As a result of the health and education sector reforms, being implemented by the majority of countries in the Americas, Health Promotion strategies are now being implemented in the school setting, thus creating new opportunities for the implementation of comprehensive school health programs throughout the Region.

The HPS Regional Initiative proposes the use of health promotion strategies that apply theories, models, and tools with solid scientific bases. PAHO/WHO formally launched the Initiative in 1995, in response to countries' needs and priority for comprehensive and sustainable school health programs, and as a commitment to Health Promotion in the school setting [71]. The Initiative is based on a comprehensive conceptual framework, with a multidisciplinary and multisectoral approach that considers people in the context of their daily life, within their family, their community, and their society.

HPS promote the development of knowledge, abilities, and skills to allow individuals to care for their health and that of others, to minimize risk behaviors and especially to adopt and maintain healthy lifestyles [72]. The Initiative contributes to the establishment of equitable social gender relationships, encouraging civic spirit and democracy, and strengthening the traditions of solidarity and community participation. It advocates for the promotion and protection of human rights and fundamental freedoms in schools and surrounding communities. As such, also contributes to the MDGs.

A regional survey in 19 Latin American countries [73] showed that 94% of the countries were developing the HPS strategy. In almost all cases (90%), the HPS strategy is being implemented in public primary schools in urban areas. 82% of the countries have school health plans predominantly in primary schools. 94% of the countries have policies aimed at health promotion of the school-age population, and 82% have specific policies related to the Health-Promoting Schools strategy. 30% of the countries have designated budgets to finance school health programs. NGOs (national or local) support the financing of such activities in 71% of the cases. About one-third of the countries (29.4%) received loans or financing from international organizations to support school health programs. These data, together with other vital information from the countries, as well as information from case studies and countries' visits provided the foundation for the development of the Plan of Action 2003–2012 for the Health-Promoting Schools Regional Initiative [74].

The Health-Promoting Schools Regional Initiative is composed of three main components [74]: comprehensive health education, including Life Skills training; healthy physical and psychosocial environments; and health and nutrition services and active life. The comprehensive Health Education component, which includes Life Skills training, is directed to strengthen the capacity of children, adolescents, and youth to acquire and utilize knowledge, attitudes, values, skills, and competencies necessary to promote and protect their own health and that of their families and communities. 88% of Latin American countries include Health Education as a transversal element of their school curricula [73]. Subjects covered by the health educational activities include addictions (94%); personal hygiene, sexual and reproductive health, physical education and sports (88%); HIV/AIDS, food and nutrition, utilization of health services (82%); and self-esteem, immunizations, waste management, and life skills (70%). Most Latin American countries include physical exercise and recreation.

The creation and maintenance of healthy school settings and surrounding environments, the second component of the Initiative, must guarantee minimum conditions of safety and environmental sanitation conducive to the health, well-being, and development of the maximum potential of children and other members of the educational community. 70% of the countries have policies to prevent smoking in schools, and 64% have programs to prevent violence in the school setting. There are major disparities among the countries of the Region with regard to the number of schools with access to water and drinking water, and in at least half of the countries where this information is available, the coverage of these services is low or unsatisfactory [73].

The third component, access to health and nutrition services and active life in the school setting, aims to the development of planned and organized activities that respond to the needs and priority of students and the educational communities. 76% of countries have established guidelines about health services to be provided to the school population, which almost always include periodic medical controls, vaccination and, to a very limited extent, other interventions such as early detection of scoliosis, psychological counseling, and gynecological care [73].

Member States, under the auspices of the HPS Regional Initiative, are developing Regional Guidelines for certification and accreditation of HPS. These Guidelines will facilitate the strengthening of school health programs and activities throughout the Region to ensure the quality and sustainability of the planning, implementation, and evaluation of Health Promotion strategies in the school setting [73].

A Health-Promoting school is a school that [74]:

• Implements policies that support dignity and individual and collective well-being and offers multiple opportunities for the growth and development of children and adolescents within the context of learning and success of the school community (including educators, students, and their families);

• Implements strategies to promote and support learning and health, utilizing all means and resources available for this purpose and involving personnel from the health and education sectors and community leaders in the implementation of planned school activities (e.g., comprehensive health education and Life Skills training; strengthening of protective factors and reduction of risk behaviors; facilitation of access to school health services, nutrition, and physical education);

• Involves all members of the school and community (including teachers, parents, students, leaders and non-governmental organizations) in decision-making and the implementation of interventions to promote learning, encourage healthy lifestyles, and carry out health promotion projects in the community;

• Has an action plan to improve the physical and psychosocial school environment and surroundings (e.g., standards and regulations for school environments free from smoking, drugs, abuse, and any form of violence; access to safe drinking water and health facilities; nutrition services), trying to set a good example through the creation of healthy school environments and the implementation of activities planned outside the school setting aimed at the community;

• Implements actions to evaluate and improve the health of students, the educational community, families, and members of the community in general, and works with community leaders to ensure access to nutrition, physical activity, counseling, and health and referral services;

• Offers relevant and effective training and educational materials to educators and students; and

• Has a local committee on education and health with the active participation of parents associations, NGOs, and other organizations in the community.

### LAC network of Health-Promoting Schools

Health-Promoting Schools Networks in LAC offer unique opportunities to continue the dialogue on health promotion and health education in all settings, and to facilitate the sharing of ideas, resources, and experiences to nurture the commitment and enthusiasm of school health personnel and experts dedicated to improving Health Promotion programs and activities in the school setting.

The Latin American Network of Health-Promoting Schools originated at the First Meeting of the Network, in 1996 in San José, Costa Rica [75]. The second meeting was held in Mexico in 1998; the third meeting in Quito, Ecuador, in 2002 [76]; and the fourth meeting in San Juan, Puerto Rico in 2004 [77].For the Caribbean countries, the first constitutive meeting of the Caribbean Network of Health-Promoting Schools was held in 2001, in Bridgetown, Barbados [78]. As of 2002, 29% of the countries had created national networks of HPS. All Latin American countries and 14 Caribbean countries are currently participating in the LAC Networks of Health-Promoting Schools.

### Planned strategies for strengthening Health-Promoting Schools in the Americas

Member States, under the auspices of PAHO/WHO, have defined six major strategies and pertinent lines of action for the period 2003–2012 [74]. The six strategies and respective lines of action for 2003 to 2012, channeled through the three components of the Health-Promoting Schools, are firmly supported by healthy public policies that facilitate the implementation of school health programs and activities that aim to sustainable human development. The six strategies are:

1. Advocacy for comprehensive school health programs and the Health-Promoting Schools

2. Institutionalization of the Health-Promoting Schools strategy and formulation of healthy public policies in the educational communities

3. Strengthening participation of key actors in the management of school health programs

4. Strengthening the capacity of Member States to manage the Health-Promoting Schools Initiative

5. Research, evaluation, and surveillance systems for the development of comprehensive school health programs

6. Mobilization of resources

In summary, as a result of the health and education sector reforms being implemented by the majority of the countries in the Americas, Health Promotion strategies are now being implemented in the school setting, thus creating new opportunities for the creation of comprehensive school health programs throughout the Region. The HPS regional initiative offers an important entry-point for the ND agenda, through the Education and Health sectors, which can strengthen countries' capacity for the planning and implementation of comprehensive school health programs, such as Health-Promoting Schools, which will facilitate the processes for addressing ND, including deworming programs in the school setting.

## VI. Primary Environmental Care Strategy

The most pressing environmental health problems today, in terms of deaths and illness worldwide, are those associated with poor households and communities in the developing world [79]. In rural areas and in the peri-urban slums and shanty-towns of the developing world, inadequate shelter, overcrowding, lack of clean water and sanitation, contaminated food, and indoor air pollution are by far the greatest environmental threats to human health [80]. The outcome of these threats becomes abundantly clear in the high rates of infectious disease and disability that developing communities face.

Primary Environmental Care (PEC) combines the original strategy proclaimed at Alma-Ata of primary health care and the conception of integral rural development that emerged from the agrarian policies of Third World countries during the 1970s. Within the renewed goal of health for all in the 21st century, the PEC strategy may be considered as all those actions necessary to improve and protect the local surroundings through foresight and prevention of possible problems, with tasks institutionalized at the local level [81].

According to WHO and the World Bank, environmental improvements at the household and community level would make the greatest difference for global health [82]. Specifically, the World Bank has calculated that improvements in local environmental conditions facing the poor could lower the incidence of disease by up to 40% [82].

### Agenda 21

In the 1992 United Nations Conference on Environment and Development, 179 governments adopted Agenda 21, a comprehensive plan of action that concerns all human actions that impact the environment. It states the following: "Major adjustments are needed in agricultural, environmental, and macroeconomic policy, at both the national and international levels, in developed as well as developing countries, to create the conditions for sustainable agriculture and rural development. The major objective of sustainable agriculture and rural development is to increase food production in a sustainable way and enhance food security. This will involve education initiatives, utilization of economic incentives and the development of appropriate and new technologies, thus ensuring the stable supplies of nutritionally adequate food, access to those supplies by vulnerable groups, and production of markets; employment and income generation to alleviate poverty; and natural resource management and environmental protection" [83].

In response to Agenda 21 many countries in LAC developed national frameworks which provide for the consideration of health, environmental and sustainable development issues. In trying to work within these frameworks, it is increasingly evident that the process of integrating health with environmental determinants in sustainable development decision-making is truly an inter-sectoral task. Success depends on coordination among numerous organizations, departments and groups at the international, national, and local levels.

### Environmental sector and NDs

The environmental sector is a prime example of an area that has traditionally been disposed toward inter-sectoral action and has the ability to lower the burden due to NDs. Analysis and practice of this strategy are based on a model focused on the promotion of human beings, the environment, and social development. This operational framework encourages participation and action, thus endowing individuals, communities, and societies with the power to make decisions [81].

Initiatives that derive from an environmental paradigm allow for several entry points for a reduction in the burden of NDs. Because of the strong causal relationship between most NDs and environmental factors, PEC can increase awareness and foster integration of interventions that have strong implications for both the health and environmental sectors. It allows for stakeholders to identify key problems affecting their community and to develop sustainable solutions. The previously mentioned HMC Initiative draws heavily on a foundation of environmental considerations as primary health concerns.

Strategies that fall under the umbrella of PEC include the suppression of vector populations through the provision and storage of safe water supplies, solid waste management systems, safe and adequate sewage and excreta disposal systems, water manipulation in dams and irrigation systems for vector control, vector diversion by zooprophylaxis, and vector exclusion by improved housing [84].

### Examples of inter-sectoral environmental action

Worth noting are the experiences in basic sanitation of large and mid-sized cities. The development of these systems is not only effective with regard to health and urbanization, but also efficient and equitable [85]. The efficiency is achieved through autonomous and decentralized management of the companies that provide water supplies and sewage services, while equity results from the generalized application of progressive rates for these services and general local level participation [85], and PEC may be the vehicle to stimulate their establishment and maintenance to serve all communities.

Another area in need of inter-sectoral environmental action through PEC concerns water use. Agricultural water users must increase the efficiency of water use, as competition between this sector and urban, industrial and residential users of water resources continues to increase [15,54]. Natural resource planning and comprehensive water and natural resource management that rely on a community-based approach have proven successful in the past [54,84].

### PEC in action

In 1999, the Pan American Center for Sanitary Engineering and Environmental Sciences (CEPIS) and the PAHO/WHO Country Office in Peru, began to focus on promoting PEC as a strategy for fostering healthy municipalities and communities. A broad scope of action was designed with a series of pilot projects aimed at building local environmental-management capacity in Peru and Central America. The aim of these pilot projects was to strengthen communities resolve for recognizing and controlling environmental factors harmful to health.

By supporting programs to strengthen the environmental health agenda, PAHO also sought to strengthen the leadership and advisory capabilities of the region's health ministries and improve community mobilization and inter-sectoral coordination. This task involved bringing together Ministries of Health and Environmental Affairs at such meetings as the Special Meeting of the Health Sector of Central America (RESSCA), where plans were approved for the seven Central American countries as well as the Central American Plan for Health and Environment in Sustainable Human Development [86].

In this context, water supply, sanitation and hygiene promotion programs are seen as a cohesive agenda, directly addressing the needs of the local population. Comprehensive sanitation improvement is not possible in isolation from other sectors, and special note should be taken of the relationships among water supply, sanitation and hygiene behavior change and their synergistic impact on health, particularly in relation to NDs and dengue and diarrheal diseases in marginalized populations. The environmental conditions associated with these diseases have served to further exclude severely affected populations from the social systems constructed to safeguard health as a fundamental human right [87]. These overlooked environmental factors have been directly responsible for perpetuating the cycle of poverty and reducing the quality of life for millions of people [88], and PEC is an effective tool to promote and address attention to these environmental health problems of the poor.

## VII. Inter-Sectoral Cooperation: Health and Agriculture

### Inter-American Meeting at the Ministerial Level on Health and Agriculture (RIMSA)

The importance of collaboration and the strengthening of partnerships between the health and agriculture sectors have also been recognized by PAHO. Every two years PAHO convenes the Ministers of Agriculture and Health as key representatives to engage in and foster technical cooperation and technology transfer, at the Inter-American Meetings at the Ministerial Level on Health and Agriculture (RIMSA). In recent years the RIMSA meetings have also included the Ministries of Environment and the Ministries of Tourism.

These meetings provide efficient mechanisms for the adoption of multilateral and regional agreements, which recognize the complementary nature of health and agriculture, regarding such topics as food protection, food safety, livestock production, tourism and trade. Specifically, RIMSA serves to fortify and institutionalize inter-sectoral collaboration between the agricultural and health sectors. This entails, for example, the development of integrated food protection programs by passing international resolutions in a policy and technical forum for the Americas [89,90].

Some of the technical topics on the agenda include healthy markets, food safety legislation, food safety based market improvement, food hygiene training for consumers, food handlers, and market vendors, and promotion of rural small household production. The meetings provide an opportunity to develop regional solutions and overarching policies to deal jointly with issues that affect local and national economies. Many of the topics and subsequent resolutions have a profound impact on public health.

### Neglected zoonoses

One main theme in the 2005 RIMSA meeting was that of neglected zoonoses in neglected populations. The WHO Expert Committee defined zoonoses as "those diseases and infections which are naturally transmitted between vertebrate animals and man" [91]. Some of the neglected zoonoses include plague, yellow fever, leptospirosis, bovine tuberculosis, brucellosis, leishmaniasis, Chagas disease, schistosomiasis japonica, and taeniasis/cysticercosis (*Taenia solium*). Neglected zoonoses disproportionately affect vulnerable populations, such as the rural poor. In many of these marginalized populations agriculture and human-animal interaction represent not only a means of income generation, but a means of day to day survival for individuals, families and communities.

These diseases represent significant public health safety concerns for the global population because of growing concerns about the risk of increased transmission. In addition to direct transmission, a growing number of diseases are transmitted to humans from animal reservoirs via food consumption, animal products, and human and animal waste [92]. For example, research demonstrates high levels of cysticercosis in rural Bolivian populations due to several multi-sectoral human risk factors, including absence of sanitary facilities, poor formal education and an inability to recognize infected pork [93]. Also, in the Andean highlands of South America, research indicates that fascioliasis (infection with the trematode parasite *Fasciola hepatica*) is a highly-endemic disease, where human prevalence rates are the highest known in the world due to a multitude of sheep, cattle, pig, and donkey reservoir hosts [94].

This implies that all sectors – agricultural, health, education and veterinary – must be guided in their work by a multidisciplinary and inter-sectoral approach, with full community participation. There can be no doubt that animal health has a vital role in improving the quality of human life especially in rural populations. Per the "one health" concept, an integrated human and animal-health system for specific mobile and remote sedentary populations enhances zoonoses detection and control, and offers a novel perspective for strengthening and shaping health systems in hard-to-reach rural communities [95,96]. This inter-sectoral approach represents an excellent opportunity for building a sense of personal and community responsibility for the promotion, care, and restoration of health.

Veterinarians and public health workers frequently interact with the rural population while caring for community health and livestock needs, as this is an integral part of the rural socioeconomic structure. Through these interactions close bonds of trust can be established, not only with farmers, but with entire families and the community. These individuals are well placed to enlist community participation in a variety of veterinary and public health activities such as zoonoses control, hygiene programs, and sanitation activities.

To this end, the macro policy environment created by RIMSA has the potential to serve as an entry point to foster collaboration between the veterinary, agriculture sectors and the public health sector at the local level to address the NDs. In addition to promoting inter-sectoral collaboration in schools of veterinary medicine, some authors have called for the continuation and expansion of health-education programs to train veterinary personnel for work in other public health fields [97].

Because rural households have so many different sources of income, rural development policies must go beyond an agricultural or a singular sector approach. Instead, rural development and poverty must be addressed as part of a comprehensive integrated and inter-sectoral approach that encompasses the dynamics of rural life, taking into account the interactions between health, agriculture, animal food production, and development. The RIMSA meetings represent new opportunities at the policy level to combat the NDs as contributors to rural poverty and to foster rural development and inter-sectoral collaboration as a result of an increasingly dynamic agriculture sector, greater decentralization, and more forms of participatory governance.

## VIII. Public Health and Human Rights

Vulnerable groups often suffer discrimination within a society; among them are the victims of such NDs as leprosy and lymphatic filariasis. In addition, they are sometimes subject to inhuman or degrading treatment and restrictions regarding their freedom of movement and their right to live and work in a healthy environment. Such treatment constitutes a violation of their basic human rights and fundamental freedoms, including the right to health, understood as the right to enjoy (without discrimination) health services, facilities and goods that are available, accessible, and of good quality.

The vulnerability, powerlessness and abandonment that are frequently experienced by those living with NDs require urgent actions and strategies. Although legally protected by national and internationally recognized human rights instruments, the human rights of vulnerable populations, especially health-related rights, are often ignored. PAHO/WHO believes that understanding these rights and ensuring that they are respected according to international human rights obligations is an essential step in the treatment of epidemics, illnesses and disability and an integral part of the promotion and protection of public health and disease prevention. Thus, international human rights law is an essential tool that supports Member States with:

• The recognition, promotion and protection of the right to health and other health-related rights and fundamental freedoms, in accordance with international human rights instruments that have been ratified by PAHO/WHO Member States

• The improvement of living conditions and standards of care in health facilities and services

• The strengthening of national agencies responsible for monitoring compliance with international human rights norms (including the right to health), such as the Ombudsman offices

• The formulation/reform of health plans, policies and legislation according to international human rights norms and standards

• The collaboration with international human rights bodies such as the Inter-American Commission on Human Rights of the Organization of American States (OAS) and the UN Committee on Economic, Social and Cultural Rights and the UN Special Rapporteur on the Right to Health; particularly with regard to the full realization of the right to health in connection with other basic human rights and freedoms

• The adoption of legislative, judicial, administrative, educational and other means to promote and provide accessible primary health care, community based services, health facilities and goods

• The elimination of the stigma and discrimination associated with persons who are experiencing health problems, illnesses (including the stigmatized NDs), epidemics and disability

A more cohesive human rights strategy that is consistent with the international and regional human rights binding instruments and standards is essential to continue to develop the aforementioned actions and to formulate new initiatives on human rights according to WHO guidelines. In summary, the purposes of the aforementioned initiatives are to:

• Advance and clarify the conceptual framework of human rights law as an instrument that can be used in all aspects of PAHO's work and across the Organization in order to accomplish PAHO's mission

• Ensure consistency in approaches, messages, human rights instruments and guidelines recommended by PAHO and WHO.

• Address the human rights and fundamental freedoms of vulnerable groups (in particular the right to health and health-related rights) in connection with health problems including NDs, illnesses, disability, epidemics, and access to health services.

## Summary

The persistence of the "vicious circle" between poverty and poor health demonstrates the importance of linking the activities of the health sector with those of other sectors such as education, housing, water and sanitation, labor, public works, transportation, agriculture, environment, and industry and using human rights norms and guidelines. The challenges presented by the prevention and control of the NDs present a significant opportunity to coordinate these sectors, with the goal of strengthening partnerships. It recognizes the synergistic impact across all sectors in improving health, and maintains that none of the health problems that affect a given population can be resolved and sustained exclusively by the health services system itself.

The foundation for this approach is the recognition that health is influenced by many factors, from genetic inheritance and individual behavior, to societal and family circumstances, and the social and physical environment. The intimate connection between health and the factors outside the purview of the health sector makes it an essential priority to pursue integration and establish inter-sectoral and inter-programmatic ties in order to further health development with equity and precision.

This paper has documented several success stories in the LAC region and provided examples of several PAHO initiatives and one World Bank initiative that have highlighted the importance of integrative and inter-sectoral approaches. At the national level, there is a great need to coordinate these initiatives with macro- and sectoral-level policy (e.g., sector-wide approaches). Coordination is necessary among the different international departments and agencies and within different levels of government, in order to sustain cooperation. There is still much to be learned about how to provide incentives for this coordination to take place [98].

Coordination and cooperation will be most effective if horizontal coordination takes place at all levels (global, national, local), and particularly at the local level, which should act as the focal point for defining needs and instigating change to the regional and national governments. Ministries of Health must also improve their capacity to analyze and respond to the extra-sectoral factors driving health conditions and then offer alternatives for coordinating inter-sectoral action. This requires the adaptation or development of new technologies, the establishment of new organizational frameworks within the context of decentralization, emphasis on integration through primary care and health promotion, and the modification (growth) of the professional profile of health care workers by overall improvement of managerial, technical and political skills.

**Figure 1:**
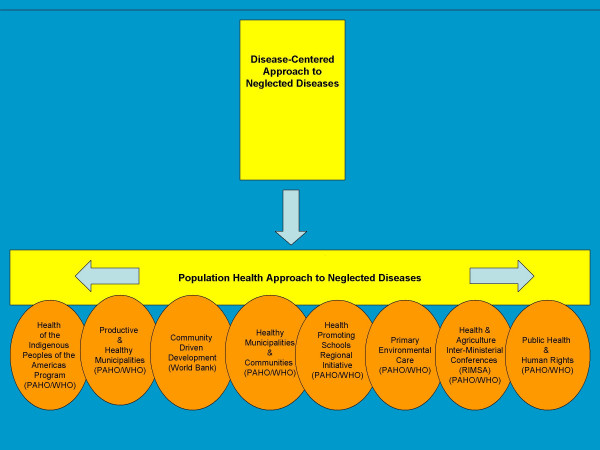
Shifting policy frameworks: an integrated, inter-sectoral approach to neglected diseases.

Refer to Figure 1 which illustrates the shift needed in policy frameworks from vertical to horizontal, integrated approaches to ND prevention, control and elimination, with an emphasis on community-level interventions. The figure highlights, as examples, the eight initiatives discussed in this document which can be incorporated with ND prevention, control and elimination.

In addition to these crucial changes within the health sector, other changes must be promoted. Public health data must be made more prominent on the national political agenda, and an effort must be made to encourage the interest and participation of other sectors in health-related matters. This, in turn, means enhancing the health sector's capacity for negotiation with the political, legislative, and budgetary sectors and the national press. In terms of policy instruments, re-channeling of government expenditures toward activities to protect and promote health for all (including neglected populations) is important, as is convincing donors to redirect their financial support toward solving environmental health problems identified through integrative and inter-sectoral efforts.

Although traditional technological tools exist to combat certain diseases and health conditions, it is the political and social commitment followed by the financial investments and innovative strategies that are necessary to take the process to a higher level. Effective sustainable development and the attainment of the MDGs is simply not possible without mechanisms related to State accountability (such as those established by human rights treaties) and a reduction in the burden of diseases that detract from worker productivity, take away educational opportunities and create chronic disability among the poorest segments of the population. Health, including its inter-sectoral and inter-programmatic dimensions, must be recognized as a crucial factor that contributes greatly to global social and economic development, as well as a fundamental right integral to the attainment of other basic human rights and liberties.

## List of Abbreviations

CDD. Community Driven Development.

CEPIS. Pan American Center for Sanitary Engineering and Environmental Sciences.

DEC. Diethylcarbamazine.

HIPA. Health of the Indigenous Peoples of the Americas.

HIV/AIDS. Human Immunodeficiency Virus/Acquired Immunodeficiency Syndrome.

HMC. Healthy Municipalities and Communities.

HPS. Health-Promoting Schools.

IMCI. Integrated Management of Childhood Illnesses.

IUCN. World Conservation Union.

IWDI. Intermittent Wet/Dry Irrigation.

LAC. Latin America and Caribbean.

MDGs. Millennium Development Goals.

NDs. Neglected Diseases.

NGO. Non-governmental Organization.

OAS. Organization of American States.

PAHO. Pan American Health Organization.

PEC. Primary Environmental Care.

PHM. Productive and Healthy Municipalities.

RESSCA. Special Meeting of the Health Sector of Central America.

RIMSA. Inter-American Meetings at the Ministerial Level on Health and Agriculture.

SAFE. Surgery, Antibiotic Therapy, Facial Cleanliness, and Environmental Improvement.

SEGEPLAN. Secretary General of Development and Planning.

STI. Sexually Transmitted Infection.

UN. United Nations.

USAID. United States Agency for International Development.

WHO. World Health Organization.

## Competing interests

The authors declare that though they are employees of the Pan American Health Organization and the World Health Organization, the contents of this paper are the sole responsibility of its authors and should not be construed as speaking for the policies of the Governing Council of the Pan American Health Organization and the World Health Organization. This paper is a contribution on the important opportunities arising from inter-sectoral and inter-programmatic dialogue to further international public health programs.

## Authors' contributions

MRP conceived the idea of the paper in collaboration with JPE, and is the principle conceptual author. JCH wrote the early drafts of the paper with editing by JPE. RR, JV, MTC, JIS, and MAG later wrote and edited their respective sections of the paper, and SKA added additional content and edited later versions of the document. All authors reviewed and approved the final version of the paper.

## Pre-publication history

The pre-publication history for this paper can be accessed here:


